# Loneliness and sarcopenia in aged care: the mediating role of fatigability

**DOI:** 10.1093/geroni/igag023

**Published:** 2026-03-18

**Authors:** Tzu-Pei Yeh, Yen-Kuang Lin, Fang-Lin Kuo, Pi-Ju Liu, Dorothy Bai, I-Hui Chen

**Affiliations:** School of Nursing, China Medical University, Taichung, Taiwan; Department of Nursing, China Medical University Hospital, Taichung, Taiwan; Graduate Institute of Athletics and Coaching Science, National Taiwan Sport University, Taoyuan, Taiwan; National Center for Geriatrics and Welfare Research, National Health Research Institutes, Yunlin, Taiwan; School of Nursing, Center on Aging and the Life Course, Purdue University, West Lafayette, Indiana, United States; School of Gerontology and Long-Term Care, College of Nursing, Taipei Medical University, Taipei, Taiwan; School of Nursing, College of Nursing, Taipei Medical University, Taipei, Taiwan

**Keywords:** Mental health, Perceived fatigability, Cross-sectional study, Health disparity, Prevalence

## Abstract

**Background and Objectives:**

Sarcopenia prevalence is higher in aged care facilities than in community settings, yet mechanistic pathways remain poorly understood. Although loneliness–sarcopenia associations are established, no studies have examined mediating mechanisms in institutional settings. We examined whether physical and mental fatigability mediate the loneliness–sarcopenia relationship in aged care residents.

**Methods:**

This cross-sectional study included 205 older adults (*M*_age_ = 80.5 ± 8.1 years; 52.2% male) from 6 aged care facilities in Taipei. Sarcopenia was assessed using the Asian Working Group for Sarcopenia 2019 criteria. Loneliness was measured via the UCLA Loneliness Scale and perceived fatigability via the validated Traditional Chinese Pittsburgh Fatigability Scale. Causal mediation analysis examined direct and indirect pathways, adjusting for demographic, health, and psychosocial confounders.

**Results:**

The sarcopenia prevalence was 58.0%. Physical fatigability significantly mediated the loneliness–sarcopenia association (odds ratio = 1.0, 95% confidence interval: 1.0-1.1), accounting for 15.0% of the total effect. The strongest mediation was for physical performance (44.2%). Substantial interaction effects between loneliness and both physical (74.7%) and mental fatigability (80.5%) suggested synergistic influences on sarcopenia risk.

**Discussion and Implications:**

Physical fatigability represents a novel, modifiable mediating pathway between loneliness and sarcopenia in aged care, with particularly strong effects on functional mobility. The substantial interaction effects support the use of combined interventions that address both psychosocial and physiological factors. Dual screening for loneliness and fatigability could identify high-risk residents, addressing the 15.0% mediation pathway. These findings inform integrated care approaches that could transform sarcopenia prevention in institutional settings. Replication in community-dwelling populations is warranted.

Innovation and Translational Significance: This study identifies physical fatigability as a novel mechanistic pathway linking loneliness to sarcopenia in aged care settings—a previously unexplored mediation relationship. The findings can be translated into practice through dual-screening protocols for loneliness and fatigability, enabling early identification of high-risk residents. The strong interaction effects (>70%) support integrated intervention designs combining social engagement with graded physical activity programs. Implementation could involve training aged care staff to recognize fatigability as an early warning sign and developing programs that simultaneously address psychosocial isolation and perceived exertion, potentially reducing sarcopenia prevalence in institutional care. These insights may inform community-based prevention strategies.

## Introduction

Sarcopenia, defined as progressive declines in skeletal muscle mass, strength, and function,^1^ represents a substantial public health burden with estimated annual healthcare costs exceeding $18.5 billion in the United States alone^2^ and affecting 10%-27% of older adults globally.^3^ Sarcopenia is closely related to, but distinct from, frailty syndrome. Although frailty represents a broader state of vulnerability encompassing multiple physiological systems, sarcopenia specifically focuses on skeletal muscle deterioration and is a key component of the physical frailty phenotype. Importantly, sarcopenia often precedes or coexists with frailty in older adults, although it can be identified and independently addressed through targeted interventions.^4^ The burden is considerably more severe among older adults in aged care facilities, where prevalence ranges from 25% to 73.7% compared with 5%-62.7% in community settings, with more profound manifestations and functional consequences.^5^^,^^6^ This heightened vulnerability reflects cumulative effects of physical inactivity, nutritional deficiencies, and higher multimorbidity burdens in institutional environments.^6^^-^^9^

Emerging evidence suggests complex relationships linking loneliness, fatigability, and sarcopenia.^10^ Loneliness—the subjective perception of insufficient companionship—affects up to 60% of aged care residents and has been directly associated with increased risks of sarcopenia.^11^^-^^13^ Studies have demonstrated that individuals with greater loneliness experience accelerated declines in muscle mass and strength, as well as physical function, likely through reduced physical activity and social participation.^14^^-^^16^ Also, loneliness may be associated with perceived fatigability in older adults.^17^

Fatigability can be conceptualized as perceived (self-reported exhaustion anchored to specific activities)^18^ or performance-based (objective declines in physical output during standardized tasks).^19^ Perceived fatigability, the focus of this study, captures the subjective experience of effort that may drive behavioral adaptations such as activity avoidance. The Pittsburgh Fatigability Scale measures perceived fatigability, encompassing both physical (effort during physical tasks) and mental (cognitive effort and exhaustion) dimensions, and offers a more objective measure than general fatigue.^20^^,^^21^ Perceived fatigability has emerged as an independent predictor of functional decline and muscle deterioration.^18^^,^^22^^,^^23^ Although associations between loneliness and sarcopenia, between loneliness and fatigability, and between fatigability and sarcopenia components have been documented, the potential mediating role of fatigability in the loneliness–sarcopenia pathway remains under-investigated. To our knowledge, this is the first study to examine fatigability as a mediator in the loneliness–sarcopenia pathway in aged care settings, addressing a critical gap in understanding the mechanistic links between psychosocial and physical health in institutionalized older adults.

Separately examining sarcopenia components is methodologically important because muscle mass and strength, and physical performance, may have distinct etiological pathways and respond differently to psychosocial exposures. Previous research suggested that loneliness may differentially affect these components through various mechanisms—for instance, physical performance may be more susceptible to behavioral changes (reduced activity), whereas muscle mass may be more influenced by metabolic and inflammatory pathways.^24^ This component-level analysis could enable identification of specific intervention targets and may provide a more-nuanced understanding of how psychosocial factors contribute to sarcopenia development.

**Figure 1:**
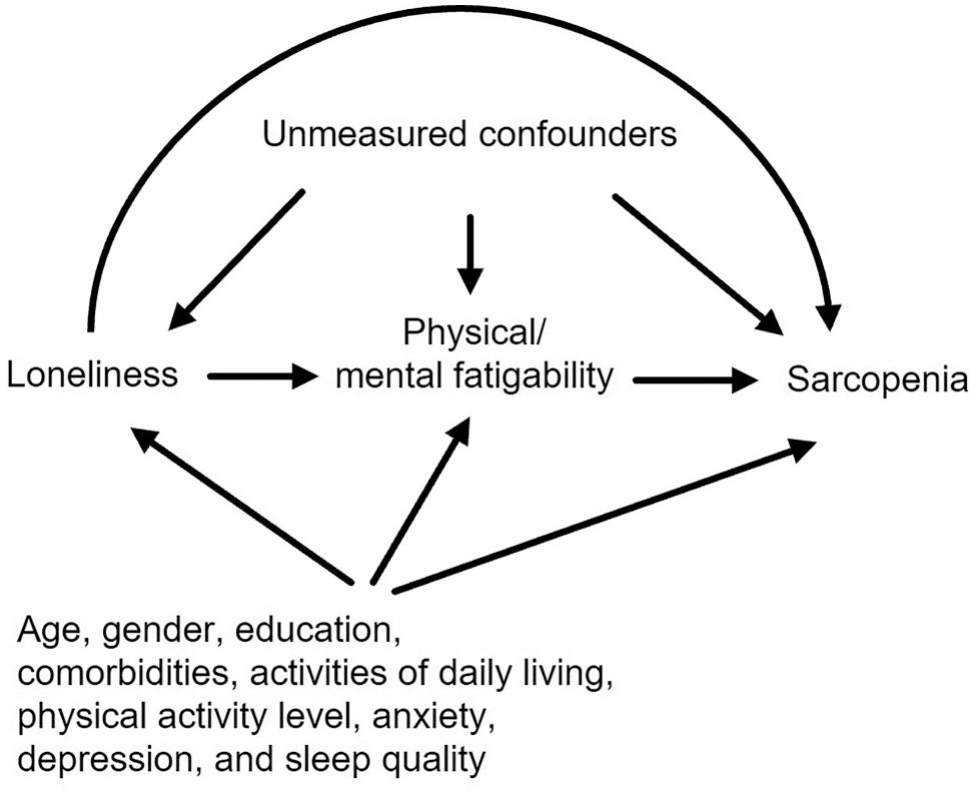
Conceptual framework and hypothesized statistical mediation pathways. Arrows represent hypothesized statistical associations examined in mediation models; cross-sectional data preclude causal inference.

Drawing on the stress process theory^25^ and the disablement process model,^26^ we hypothesized that loneliness, as a chronic psychosocial stressor, may lead to physiological and behavioral dysregulation that manifests as increased fatigability, subsequently accelerating musculoskeletal deterioration. Understanding this pathway could inform integrated interventions targeting both psychosocial and functional declines among institutionalized older adults. In this study, we therefore examined whether physical and mental fatigability mediate the relationship between loneliness and sarcopenia in older adults residing in aged care facilities (Figure 1), with potential implications for developing more-effective targeted intervention strategies.

## Methods

### Study design and participants

This cross-sectional study was conducted from October 2022 to August 2023 across 6 aged care facilities in Taipei, Taiwan, following the guidelines of Strengthening the Reporting of Observational Studies in Epidemiology (STROBE).^27^ The sample size was based on prior simulation studies, which suggested that ≥200 participants are needed to detect medium indirect effects with 80% power.^28^ Participants were recruited using convenience sampling based on existing research partnerships. Inclusion criteria were: (1) being aged ≥65 years, (2) having a Saint Louis University Mental Status examination score of ≥25, (3) having the ability to communicate in Mandarin or Taiwanese, (4) having the capacity to maintain a standing position either independently or with assistive devices, and (5) providing written informed consent. Exclusion criteria included being unable to ambulate and having a severe behavioral disorder, cognitive impairment, a terminal condition, a significant sensory impairment, or a speech disorder. A post hoc power analysis^29^ confirmed a statistical power of 0.89 to detect mediation effects at α = 0.05.

### Measures

Sarcopenia was assessed using the Asian Working Group for Sarcopenia (AWGS) 2019 criteria^30^ that evaluate 3 domains: skeletal muscle mass, muscle strength, and physical performance. Skeletal muscle mass was measured using a bioelectrical impedance analysis (InBody 430, Biospace, Seoul, Korea). The appendicular skeletal muscle mass divided by the height squared was used to derive the skeletal muscle mass index, with cutoffs of <7.0 kg/m^2^ for men and <5.7 kg/m^2^ for women. Muscle strength was assessed via a handgrip dynamometer (Takei TKK 5401, Tokyo, Japan), with low strength defined as <28 kg for men and <18 kg for women. Physical performance was evaluated using the 6-m walking test, with a speed of <1.0 m/s classified as low performance. The AWGS 2019 criteria provide sex-specific cutoffs for muscle mass and strength but do not incorporate BMI-specific adjustments, unlike some frailty phenotype definitions.^31^ We adhered to the AWGS 2019 consensus criteria, which were specifically developed and validated for Asian populations and are widely used in regional sarcopenia research. Sarcopenia was diagnosed when low muscle mass was present, accompanied by either low muscle strength or low physical performance.

Loneliness was measured using the UCLA Loneliness Scale (version 3), a validated 20-item instrument^32^ rated on a 4-point Likert scale (1 = never to 4 = often), yielding scores of 20-80, with higher scores indicating greater perceived loneliness (in this study, Cronbach’s α = 0.82). Sample items include “There is no one I can turn to” and “No one really knows me well.”

Perceived fatigability was measured using the validated Traditional Chinese version of the Pittsburgh Fatigability Scale (TC-PFS),^17^ which assesses perceived physical and mental fatigability across standardized activities.^20^^,^^21^ The 10-item scale rates anticipated fatigue levels from 0 (no fatigue) to 5 (extreme fatigue), with physical and mental fatigability scores each ranging from 0 to ∼50. Higher scores indicate greater fatigability, with established cutoffs of PFS Physical: less severe <15 and more severe ≥15; PFS Mental: less severe <13 and more severe ≥13^23^^,^^33^ (in this study, Cronbach’s α values were 0.85 for the physical and 0.80 for the mental dimension). For physical fatigability, participants rated perceived effort for activities such as “A leisurely 30-minute walk” and “Light household activities for an hour.” For mental fatigability, items included “Participating in a social activity for an hour” and “Watching television for 2 hours.”

Covariates included age, gender, educational level, chronic conditions, activities of daily living (ADLs; Barthel Index), physical activity (International Physical Activity Questionnaire)^34^ anxiety (State-Trait Anxiety Inventory)^35^ depression (15-item Geriatric Depression Scale)^36^ and sleep quality (Pittsburgh Sleep Quality Index).^37^

### Data collection and ethics

Data were collected through structured one-on-one interviews by trained research personnel using standardized protocols. Each assessment lasted approximately 50 minutes and was conducted in a private testing room with consistent conditions across facilities. The study received ethical approval from the author’s university Institutional Review Board (no. N202204055). Written informed consent was obtained from all participants.

### Statistical analysis

Data were analyzed using IBM SPSS Statistics version 28.0 (Armonk, NY, USA), SAS version 9.4 (Cary, NC, USA), and R 4.5.0 (Vienna, Austria). All variance inflation factors for physical fatigability, mental fatigability, and covariates were <5, indicating no problematic multicollinearity. A multistep approach examined associations among loneliness, fatigability, and sarcopenia. Given the cross-sectional design, our causal mediation analysis estimated statistical mediation effects under a counterfactual framework rather than through establishing temporal causality. The term “causal” refers to the analytical approach (counterfactual-based decomposition of effects) rather than claims of causal inference, which would require longitudinal data. A logistic regression was used to evaluate associations between predictor variables and binary outcomes. For the binary outcome of overall sarcopenia, odds ratios (ORs) are reported from logistic regression models. For continuous sarcopenia components (muscle mass, muscle strength, and physical performance measured as gait speed), linear regression models were employed, and regression coefficients (*B*) are reported to preserve the interpretability of unit changes in these outcomes. Causal mediation analyses using the counterfactual framework (SAS procedure CAUSALMED) were used to estimate: (1) the total effect (TE) of loneliness on sarcopenia outcomes; (2) the controlled direct effect (CDE) and natural direct effect (NDE), reflecting effects not mediated by fatigability; (3) the natural indirect effect (NIE), representing the effect mediated through fatigability; (4) the percentage mediated (NIE as a proportion of TE); and (5) the percentage attributable to the interaction, indicating synergistic effects between loneliness and fatigability. ADLs were included as a covariate to account for baseline functional status, which may influence both exposure and outcome variables. Although ADLs share conceptual overlap with physical performance, we retained this adjustment because (1) ADLs reflect the capacity for self-care activities rather than objective walking speed, (2) our primary outcome was the composite sarcopenia diagnosis rather than physical performance alone, and (3) excluding ADLs in the sensitivity analyses did not substantively alter the main findings. All models were adjusted for covariates, with significance set at *p *< .05. Sensitivity analyses assessed potential influences of unmeasured confounding variables.^38^

## Results

### Participant characteristics

The sample comprised 205 older adults (mean age = 80.5 ±8.1 years; 52.2% male) who generally had low educational attainment (40.5% had completed elementary school or less). Participants averaged 2.6 ± 1.8 chronic conditions and scored 76.9 ± 6.8 on the Barthel Index (Table 1). Most (59.5%) reported low physical activity levels. Mean scores were: state anxiety 30.3 ± 10.3, trait anxiety 32.4 ± 10.8, depression 4.0 ± 3.5, sleep quality 8.3 ± 3.4, loneliness 46.9 ± 10.0, physical fatigability 26.2 ± 11.1, and mental fatigability 23.2 ± 12.3. Based on AWGS 2019 criteria, 58.0% of participants had sarcopenia. Based on established cutoffs, the majority of participants exhibited more severe physical fatigability (*n *= 165, 80.5%) and more severe mental fatigability (*n *= 151, 73.7%).

**Table 1:** Basic information of participants (*N *= 205).

Variable	Mean (*SD*)	*n* (%)
**Age (years)**	80.5 (8.1)	
**Gender**		
Male		107 (52.2)
Female		98 (47.8)
**Education**		
Illiterate		15 (7.3)
Elementary school		68 (33.2)
Junior high school		35 (17.1)
Senior high school		48 (23.4)
University and above		39 (19.0)
**Number of chronic diseases**	2.6 (1.8)	
**Activities of daily living**	76.9 (6.8)	
**Physical activity level**		
Low		122 (59.5)
Moderate		82 (40.0)
High		1 (0.5)
**Anxiety**		
State	30.3 (10.3)	
Trait	32.4 (10.8)	
**Depression**	4.0 (3.5)	
**Sleep quality**	8.3 (3.4)	
**Loneliness**	46.9 (10.0)	
**Fatigability**		
Physical score	26.2 (11.1)	
More severe (≥15)		165 (80.5)
Less severe (<15)		40 (19.5)
Mental score	23.2 (12.3)	
More severe (≥13)		151 (73.7)
Less severe (<13)		54 (26.3)
**Sarcopenia**		119 (58.0)
Muscle mass		
Male	6.7 (2.7)	
Female	5.5 (2.0)	
Muscle strength		
Male	20.3 (7.5)	
Female	13.4 (4.6)	
Physical performance	0.7 (0.3)	

Abbreviation: *SD* = standard deviation.

### Mediation analysis

#### Effects on overall sarcopenia

Physical fatigability significantly mediated the association between loneliness and sarcopenia (Table 2). The TE of loneliness on sarcopenia was significant (odds ratio [OR] = 1.3, 95% confidence interval [CI]: 1.2-1.5, *p *< .001). The NIE via physical fatigability was also significant (OR = 1.0, 95% CI: 1.0-1.1, *p *= .019), accounting for 15.0% of the TE (*p *= .006). The NDE remained significant (OR = 1.3, 95% CI: 1.2-1.4, *p *< .001), indicating partial mediation.

**Table 2:** Mediation analysis of physical and mental fatigability in the relationship between loneliness and sarcopenia.

Outcome variable	Estimate	*SE*	95% CI	*z*	*p*	% Mediated (*SE*)	*p*
**Sarcopenia (binary outcome)**							
***Physical fatigability as a mediator***							
Total effect	1.3^a^	0.1	1.2, 1.5	4.5	<.001	—	—
Controlled direct effect	1.3^a^	0.1	1.2, 1.4	4.4	<.001	—	—
Natural direct effect	1.3^a^	0.1	1.2, 1.4	4.4	<.001	—	—
Natural indirect effect	1.0^a^	0.02	1.0, 1.1	2.3	.019	15.0 (5.5)	.006
Percentage due to interaction	74.7	9.4	56.2, 93.2	7.9	<.001	—	—
***Mental fatigability as a mediator***							
Total effect	1.3^a^	0.1	1.2, 1.5	4.5	<.001	—	—
Controlled direct effect	1.3^a^	0.1	1.2, 1.4	4.5	<.001	—	—
Natural direct effect	1.3^a^	0.1	1.2, 1.4	4.5	<.001	—	—
Natural indirect effect	1.0^a^	0.02	1.0, 1.1	1.5	.139	9.3 (5.7)	.103
Percentage due to interaction	80.5	9.3	62.2, 98.8	8.6	<.001	—	—
**Sarcopenia components**							
**Muscle mass**							
***Physical fatigability as a mediator***							
Total effect	−0.1^b^	0.02	−0.1, −0.1	−6.4	<.001	—	—
Controlled direct effect	−0.1^b^	0.01	−0.1, −0.1	−5.8	<.001	—	—
Natural indirect effect	−0.02^b^	0.01	−0.04, −0.004	−2.5	.013	20.7 (7.3)	.005
***Mental fatigability as a mediator***							
Total effect	−0.1^b^	0.02	−0.1, −0.1	−6.4	<.001	—	—
Controlled direct effect	−0.1^b^	0.01	−0.1, −0.1	−5.8	<.001	—	—
Natural indirect effect	−0.02^b^	0.01	−0.04, −0.004	−2.5	.013	20.7 (7.3)	.005
**Muscle strength**							
***Physical fatigability as a mediator***							
Total effect	−0.2^b^	0.04	−0.2, −0.1	−3.7	<.001	—	—
Controlled direct effect	−0.1^b^	0.04	−0.2, −0.04	−3.0	.003	—	—
Natural indirect effect	−0.04^b^	0.02	−0.1, −0.01	−2.3	.019	24.1 (10.4)	.020
***Mental fatigability as a mediator***							
Total effect	−0.2^b^	0.04	−0.2, −0.1	−3.7	<.001	—	—
Controlled direct effect	−0.1^b^	0.04	−0.2, −0.1	−3.4	<.001	—	—
Natural indirect effect	−0.02^b^	0.02	−0.1, 0.01	−1.5	.100	16.2 (10.0)	.107
**Physical performance**							
***Physical fatigability as a mediator***							
Total effect	−0.004^b^	0.002	−0.01, −0.001	−2.4	.015	—	—
Controlled direct effect	−0.002^b^	0.002	−0.01, 0.001	−1.5	.142	—	—
Natural indirect effect	−0.002^b^	0.001	−0.003, 0.0	−2.4	.015	44.2 (20.2)	.029
***Mental fatigability as a mediator***							
Total effect	−0.004^b^	0.002	−0.01, −0.001	−2.4	.015	—	—
Controlled direct effect	−0.003^b^	0.002	−0.01, 0.0	−1.9	.053	—	—
Natural indirect effect	−0.001^b^	0.001	−0.003, 0.0	−1.5	.131	29.1 (17.5)	.096

All models were adjusted for age, gender, education, comorbidities, activities of daily living, physical activity, anxiety, depression, and sleep quality. Statistically significance at *p *< .05.

Abbreviations: CI = confidence interval; *SE* = standard error.

a Values are presented as odds ratios (ORs).

b Values are presented as regression coefficients.

Mental fatigability did not significantly mediate the loneliness–sarcopenia relationship (NIE: OR = 1.0, 95% CI: 1.0-1.1, *p *= .139). However, the percentage of TE attributable to the interaction was substantial for both mental (80.5%, 95% CI: 62.2%-98.8%, *p *< .001) and physical fatigability (74.7%, 95% CI: 56.2%-93.2%, *p *< .001), suggesting strong synergistic effects.

#### Effects on sarcopenia components

Both physical and mental fatigability significantly mediated the association between muscle mass and loneliness (TE: *B* = −0.1, 95% CI: −0.1, −0.1, *p *< .001; NIE: *B* = −0.02, 95% CI: −0.04, −0.004, *p *= .013), each accounting for 20.7% of the TE (*p *= .005). For muscle strength, only physical fatigability demonstrated significant mediation (TE: *B* = −0.2, 95% CI: −0.2, −0.1, *p *< .001; NIE: *B* = −0.04, 95% CI: −0.1, −0.01, *p *= .019), accounting for 24.1% of the TE (*p *= .020). For physical performance, physical fatigability significantly mediated the relationship (TE: *B* = −0.004, 95% CI: −0.01, −0.001, *p *= .015; NIE: *B* = −0.002, 95% CI: −0.003 to 0.0, *p *= .015), accounting for 44.2% of the TE (*p *= .029), with the CDE becoming nonsignificant (*B* = −0.002, 95% CI: −0.01, 0.001, *p *= .142), indicating full mediation.

### Sensitivity analysis

Sensitivity analyses (see Supplementary Figure 1 for a color version of this figure) confirmed the robustness of our findings. The mediation effect of physical fatigability on the loneliness–sarcopenia relationship remained statistically significant across a reasonable range of sensitivity parameter (ρ) values, suggesting that our results were relatively insensitive to unmeasured confounding. For mental fatigability, sensitivity analyses revealed that mediation effects approached significance for muscle mass (ρ values between −0.3 and 0.3) but remained nonsignificant for overall sarcopenia across the tested range of sensitivity parameters (see Supplementary Figure 1B, F-H for a color version of this figure). These findings suggest that although mental fatigability may play a role in specific sarcopenia components, its mediating effect is less robust to potential unmeasured confounding compared to physical fatigability.

## Discussion

Results of this study reveal complex interrelationships among loneliness, fatigability, and sarcopenia among older adults in aged care settings. Using a causal mediation analysis, we identified physical fatigability as a significant mediator in the pathway linking loneliness to sarcopenia.

### Physical fatigability as a key mediator

Identifying physical fatigability as a significant mediator in the relationship between loneliness and sarcopenia represents a key contribution to understanding the multifactorial etiology of sarcopenia in older adults. Our study extends the existing literature by identifying physical fatigability as a previously unexplored mechanistic pathway, providing the first empirical evidence of its mediating role in institutionalized populations. This finding is consistent with theoretical frameworks that posit that loneliness induces physiological dysregulation—including heightened sympathetic nervous system activity and activation of the hypothalamic-pituitary-adrenal (HPA) axis—that may promote muscle catabolism.^39^ Our results extend this framework by establishing physical fatigability as a mechanistic pathway through which loneliness may exert its deleterious effects on muscle health. The quantifiable mediation effect (15.0% for overall sarcopenia and 44.2% for physical performance) provides specific targets for intervention development and evaluation. The substantial interaction between loneliness and physical fatigability suggests a synergistic effect, whereby their co-occurrence significantly magnifies the risk of sarcopenia beyond the additive impact of each factor alone. In practical terms, this indicates that lonely individuals who also experience more severe physical fatigability may face an elevated risk of muscle decline due to increased perceived exertion, leading to avoidance of physical activity and consequent deconditioning.^40^^,^^41^ This cycle may reinforce inactivity and accelerate sarcopenic progression in institutionalized older populations.

Physical fatigability fully mediated the association between loneliness and physical performance, a particularly salient finding given that physical performance reflects the functional consequences of muscle loss and is strongly predictive of adverse health events in later life.^42^ The finding of full mediation implies that physical fatigability may be the principal conduit through which loneliness compromises functional mobility. Additionally, physical fatigability partially mediated the effects of loneliness on muscle strength, indicating the presence of other contributing mechanisms. For muscle mass, both physical and mental fatigability exhibited mediating effects, reinforcing sarcopenia’s multidimensional nature.^43^

This hierarchical pattern of mediation suggests a plausible trajectory: loneliness first impairs perceived energy levels, altering physical activity behaviors and ultimately leading to observable muscular deterioration. These findings support and expand on previous evidence that fatigability predicts mobility decline in older adults,^44^ and offer new insights by linking this process to modifiable psychosocial exposures.

Notably, participants exhibited markedly elevated fatigability scores (physical: 26.2 ± 11.1; mental: 23.2 ± 12.3), substantially exceeding the established thresholds for more severe fatigability (PFS physical ≥15; PFS mental ≥13). Indeed, 80.5% of participants met criteria for more severe physical fatigability and 73.7% for more severe mental fatigability. These proportions substantially exceed those reported in community-dwelling older adults using the same PFS cutoffs—ranging from 41% to 66% for physical fatigability and 22% to 42% for mental fatigability across major aging cohorts^18^—underscoring the disproportionate fatigability burden in institutional care settings. This high baseline fatigability reflects the vulnerable health status of aged care residents and may have influenced our findings in several ways. First, ceiling effects could have attenuated mediation estimates if many participants were already at maximum fatigability levels. Second, the restricted range of fatigability scores may have limited our ability to detect linear dose-response relationships. Third, these elevated prevalences indicate that the majority of residents would qualify for intervention based on established cutoffs, supporting the feasibility and reach of fatigability-focused programs in this setting. Future studies in populations with greater variability in fatigability may reveal stronger or different mediation patterns.

### Contextual role of mental fatigability

Although mental fatigability did not significantly mediate the relationship between loneliness and overall sarcopenia in the primary analyses, strong interaction effects suggested its potential contextual importance. Mental fatigability might not act as an independent mediator but may exacerbate the influence of loneliness on sarcopenia when both factors co-occur, indicating a possible synergistic interaction rather than a straightforward causal pathway.

Divergent results between the primary and sensitivity analyses underscore the complexity of this relationship. Although mental fatigability did not meet statistical thresholds for mediation in the primary models, sensitivity analyses revealed potential mediation effects for specific sarcopenia components. These inconsistencies may be attributable to methodological differences in model assumptions or could reflect a more nuanced role of mental fatigability that is not readily captured by a mediation analysis—particularly in the context of institutional care, where cognitive and social stimulation are often constrained. It is also possible that mental fatigability exerts delayed or cumulative effects on physical health that are not readily observable in cross-sectional data. Prior research suggested that psychological factors, including cognitive exhaustion and reduced mental resilience, may influence health outcomes over time through indirect behavioral and physiological mechanisms.^45^ This temporal dimension may explain the absence of strong mediation effects in point-in-time analyses while supporting its relevance in longitudinal trajectories of functional decline.

### Biological and behavioral mechanisms

The significant CDE observed between loneliness and sarcopenia suggests that loneliness contributes to sarcopenia risk through mechanisms beyond the fatigability pathway. This finding aligns with prior evidence indicating that loneliness can independently trigger dysregulation of neuroendocrine and immune responses, including heightened HPA axis activity, sympathetic nervous system arousal, and elevated systemic inflammation.^46^^-^^48^ These physiological changes may accelerate muscle protein breakdown and impair anabolic processes, directly influencing muscle metabolism and contributing to sarcopenic decline.

Our mediation findings imply that such biological disruptions may also manifest behaviorally as increased fatigability, which in turn limits physical function. However, the persistence of a significant DE even after accounting for fatigability highlights the multifactorial nature of sarcopenia and supports the hypothesis that loneliness can affect muscle health via both physiological and behavioral routes.

The interaction effects observed between loneliness and fatigability suggest a complex pathophysiological process wherein these factors may potentiate each other’s impact on muscle health. For instance, loneliness-induced inflammatory responses may enhance perceptions of effort during physical activities, whereas elevated fatigability may reinforce social withdrawal and exacerbate feelings of loneliness. This bidirectional relationship creates a potentially detrimental cycle that may be particularly difficult to interrupt once established, underscoring the importance of early and multifaceted intervention approaches in aged care contexts.

### Implications

These findings have important implications for sarcopenia prevention. Identifying physical fatigability as a significant mediator in the loneliness–sarcopenia pathway suggests that interventions aimed at reducing perceived effort during physical activity may help attenuate the adverse effects of loneliness on muscle health. Such interventions could include progressive resistance training with appropriate intensity modulation to enhance exercise tolerance,^49^ cognitive-behavioral strategies addressing effort perceptions,^50^ and techniques to increase motivation for physical activity among socially isolated residents.

The strong interaction effects observed between loneliness and both physical and mental fatigability further underscore the importance of multidimensional approaches that address both psychosocial and functional domains of aging. Addressing loneliness in isolation may be insufficient for residents experiencing high fatigability, and vice versa. Integrated programs combining social engagement with structured physical activity—such as group-based or peer-supported exercise interventions—may be particularly effective in mitigating sarcopenia risk among lonely older adults.^51^

From a practical implementation perspective, aged care facilities could adopt a staged approach beginning with incorporating brief screening for both loneliness and fatigability into routine quarterly assessments. Facilities could then develop risk stratification protocols using established cutoffs from the literature, prioritizing residents with concurrent more severe loneliness and elevated physical fatigability for intensive interventions, although specific thresholds may need to be adjusted based on facility populations. This would be supported by training existing staff through brief in-service sessions to distinguish fatigability from general fatigue, focusing on recognizing activity-specific exhaustion patterns rather than overall tiredness. Residents identified as simultaneously experiencing both conditions may represent a high-risk subgroup warranting tailored interventions, such as peer pairing based on functional abilities, to simultaneously reduce social isolation and promote activity engagement. Environmental modifications could be systematically implemented, such as creating “fatigability-friendly” exercise spaces with built-in rest areas, visual progress markers, and social interaction zones that normalize pacing and rest breaks during activities.

The differential mediation patterns across sarcopenia components point to the need for personalized interventions. Older adults who present with distinct sarcopenia profiles (e.g., impaired physical performance vs low muscle mass) may benefit from targeted strategies that address the most relevant mediating pathways. For example, those with primarily performance-related deficits might benefit most from interventions focused on reducing physical fatigability and enhancing activity tolerance, while those with primarily mass-related deficits might require attention to both physical and mental fatigability components.

From a policy perspective, our findings support the development of care standards that recognize the interconnectedness of psychosocial well-being and physical function. Resource allocation should prioritize integrated approaches rather than siloed interventions that address either loneliness or physical function in isolation. Training programs for aged-care staff should emphasize recognition of both loneliness and fatigability as early warning signs for potential sarcopenic progression, enabling timely preventive measures.

Although a formal economic analysis was beyond this study’s scope, the high sarcopenia prevalence (58.0%) and identified mediation pathway suggest that integrated interventions could potentially offer resource efficiency by addressing a pathway that accounts for 15.0% of sarcopenia risk, supporting the value of dual-domain approaches over single-focus interventions.

Although this study focused on aged care residents, the identified fatigability-mediated pathway may have broader relevance for community-dwelling older adults. Loneliness and fatigability are prevalent in community settings as well, although typically at lower levels than in institutional populations. Community-based interventions targeting social engagement and activity tolerance—such as walking groups or community centers offering graduated exercise programs—could potentially interrupt this pathway before institutional care is needed. However, the magnitude of mediation effects may differ in community populations with higher baseline physical function in general, warranting replication in non-institutional samples.

### Future directions

Our findings point to several important avenues for future research. Longitudinal studies are needed to establish temporal sequences among loneliness, fatigability, and sarcopenia components and determine whether changes in fatigability mediate the relationship between changes in loneliness and subsequent sarcopenia progression. Such designs would strengthen causal inferences and provide insights into the dynamic interplay of these factors over time. Future research should also examine whether fatigability-mediated pathways operate consistently across different residential settings and risk factors.^52^ Intervention studies specifically targeting the loneliness–fatigability pathway should be developed and evaluated. These might include combined approaches that simultaneously address social connectedness and perceived exertion, such as socially engaging exercise programs with progressive intensity adaptation. Formal cost-effectiveness analyses are needed to quantify the economic benefits of integrated versus single-domain interventions in aged care settings.

Further exploration of biological mediators is warranted, including inflammatory markers, stress hormones, and metabolic factors that may link psychosocial stress to muscle deterioration. Incorporation of such biomarkers alongside behavioral and self-reported measures would strengthen the empirical basis for the proposed pathophysiological model and potentially identify additional intervention targets. Sex-specific analyses should be conducted to determine whether the mediating role of fatigability differs between older men and women, given known sex differences in muscle physiology, loneliness experiences, and activity patterns in later life.^53^ Such analyses may reveal important nuances for tailoring interventions to specific demographic subgroups within aged care settings. Finally, implementation research is needed to identify effective strategies for integrating psychosocial and physical assessments and interventions into routine aged-care practice. This includes developing practical screening protocols, training staff in recognition and response, and designing sustainable intervention approaches that can be maintained within existing care structures and resource constraints.

### Limitations

Several limitations of this study warrant consideration. First, the cross-sectional design precludes causal inferences regarding temporal sequences among loneliness, fatigability, and sarcopenia. Second, despite adjusting for multiple confounders, residual confounding remains possible. Specifically, data on nutritional status, inflammatory biomarkers, and stress-related hormones were not collected; including these variables could provide further insights into the biological mechanisms underpinning the observed relationships. Third, both loneliness and fatigability were assessed using self-reported instruments, which may have been subject to recall bias and individual variability in interpretation. Fourth, the inclusion of ADLs as a covariate may have introduced some degree of overadjustment given their correlation with physical performance; however, sensitivity analyses excluding ADLs yielded similar results. Fifth, the use of sex-specific but not BMI-adjusted cutoffs for physical performance may have introduced measurement variation. Sixth, convenience sampling limited the generalizability and may have introduced selection bias. Lastly, the timing of data collection coincided with the post-acute phase of the COVID-19 pandemic, during which aged care facilities were transitioning back to routine operations. The psychological effects of prolonged isolation during the earlier phases of the pandemic may have influenced participants’ perceptions of loneliness and fatigability.

## Conclusions

In this study, we identified physical fatigability as a significant statistical mediator in the relationship between loneliness and sarcopenia of residents of aged care facilities, with the particularly strongest effects on physical performance. The substantial interaction effects suggest that combined interventions addressing both loneliness and fatigability may be most effective for sarcopenia prevention in aged care settings. Although these findings require replication in community-dwelling populations, they contribute to a more comprehensive understanding of biological and behavioral mechanisms of sarcopenia and highlight promising targets for interventions tailored to the specific needs and challenges of residential aged care populations.

## Supplementary Material

igag023_Supplementary_Data

## Data Availability

De-identified data supporting the study conclusions are available from the corresponding author upon reasonable request and appropriate ethical approval.
