# Supplementary material for: Loneliness and sarcopenia in aged care: the mediating role of fatigability
Source: Innov Aging. 2026 Mar 18;10(6):igag023. doi: 10.1093/geroni/igag023 (PMC13166890; doi:10.1093/geroni/igag023)
Supplement: igag023_Supplementary_Data [file igag023_supplementary_data.docx]

***Innovation in Aging* Supplementary Material: Loneliness and sarcopenia in aged care: The mediating role of fatigability.**

**Supplementary Figure 1.** Sensitivity analysis for causal mediation pathways examining how loneliness affects sarcopenia and its components through fatigability.

| 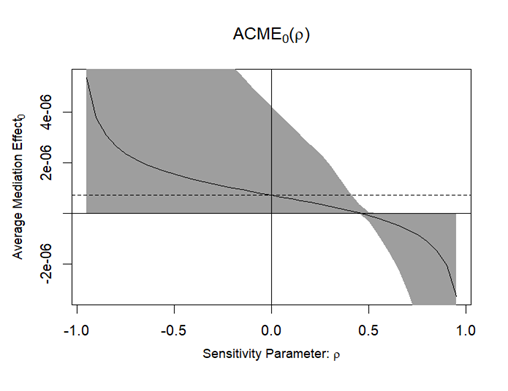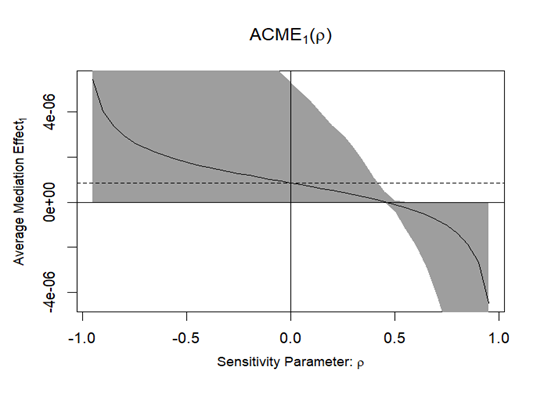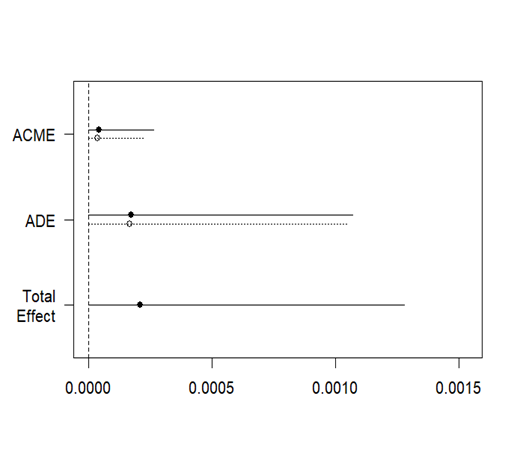 |
| --- |
| A. Loneliness 🡪 physical fatigability 🡪 sarcopenia |
| 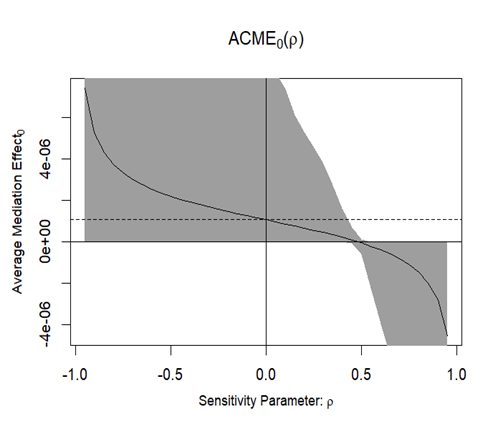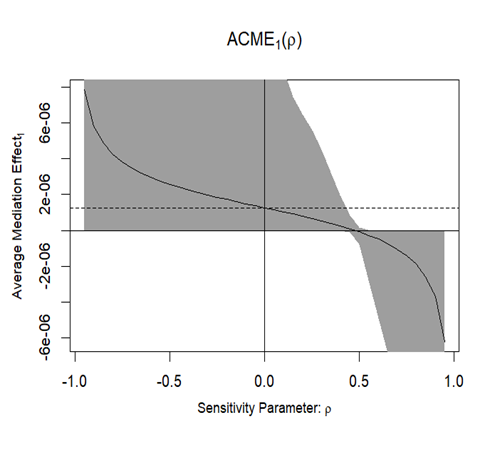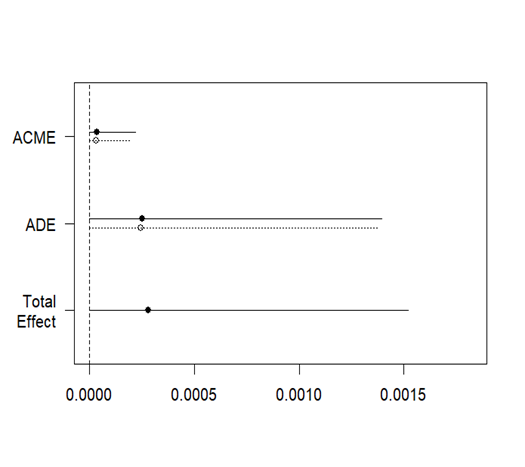 |
| B. Loneliness 🡪 mental fatigability 🡪 sarcopenia |

| 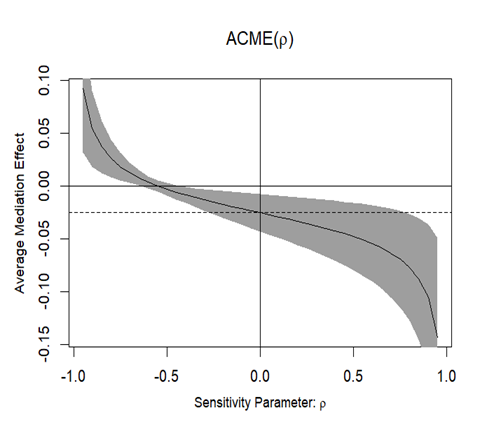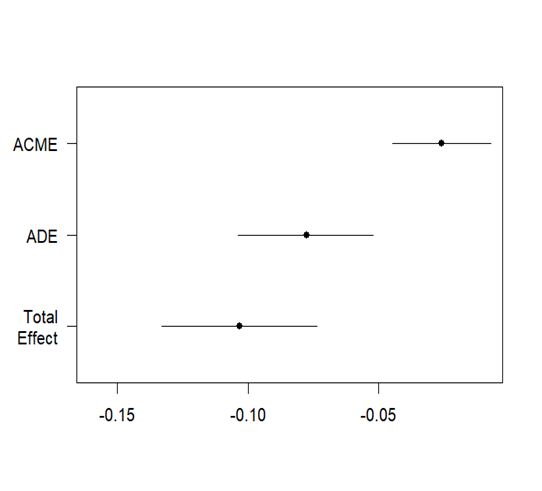 |
| --- |
| C. Loneliness 🡪 physical fatigability 🡪 muscle mass |
| 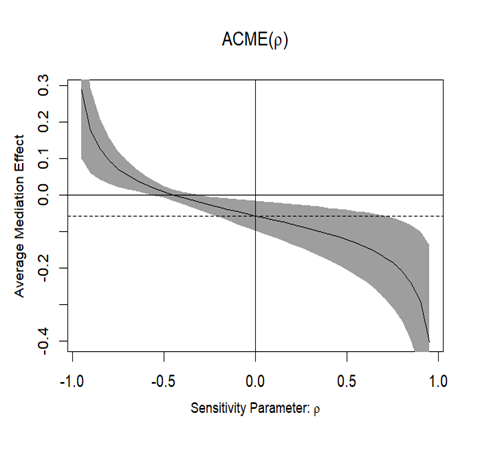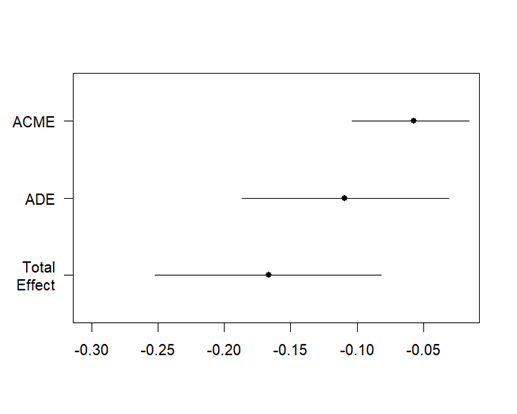 |
| D. Loneliness 🡪 physical fatigability 🡪 muscle strength |
| 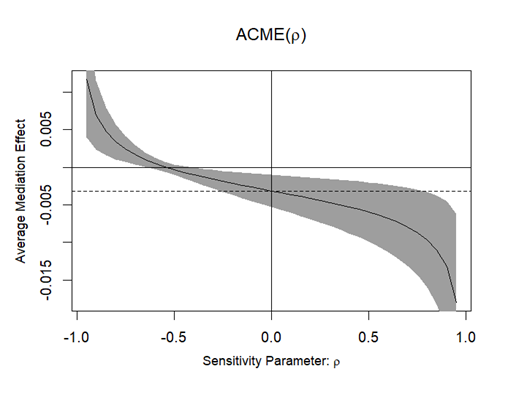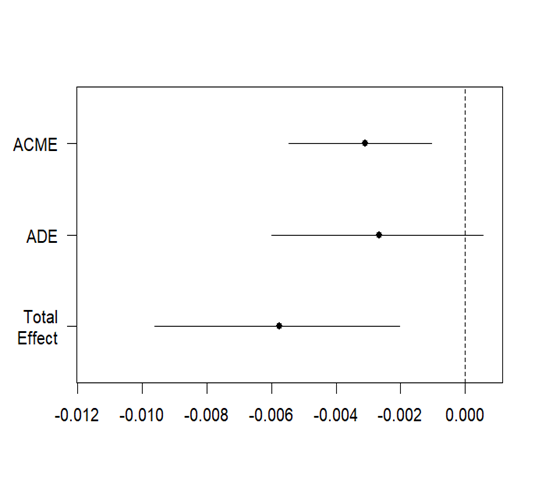 |
| E. Loneliness 🡪 physical fatigability 🡪 physical performance |
| 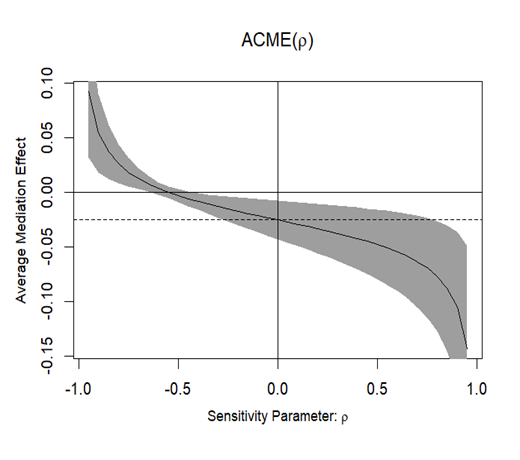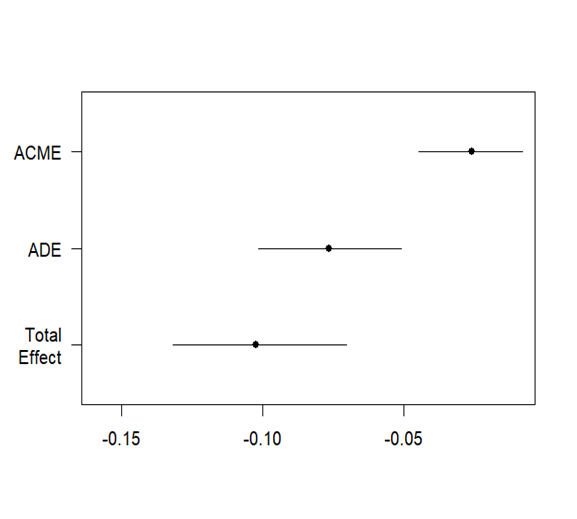 |
| F. Loneliness 🡪 mental fatigability 🡪 muscle mass |
| 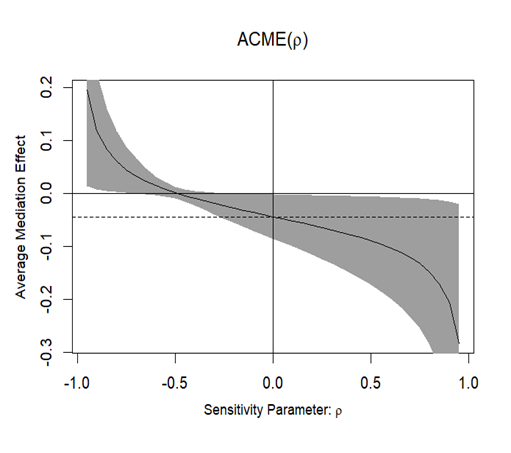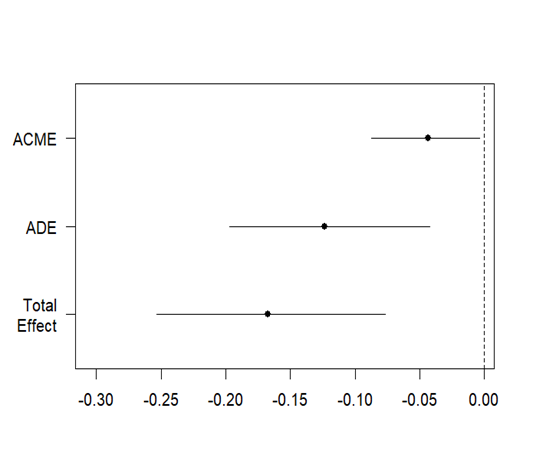 |
| G. Loneliness 🡪 mental fatigability 🡪 muscle strength |
| 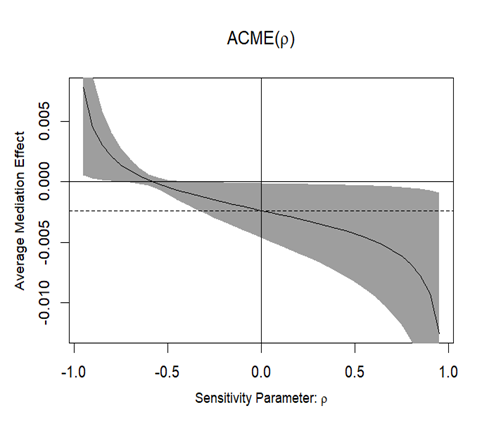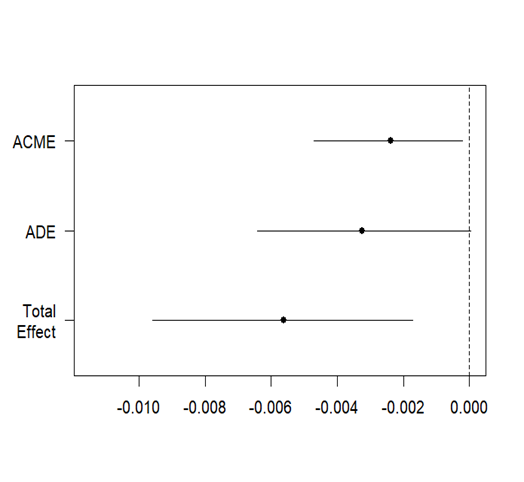 |
| H. Loneliness 🡪 mental fatigability 🡪 physical performance |

**Note.** This figure presents sensitivity analyses examining robustness to unmeasured confounding. The sensitivity parameter, ρ, represents the correlation between unmeasured confounders affecting both the mediator (fatigability) and the outcome (sarcopenia); ρ=0 indicates no unmeasured confounding, while values approaching ±1 indicate strong confounding. Left panels show how the average causal mediation effect (ACME) changes across ρ values, with the gray shaded region representing 95% confidence intervals. Right panels display point estimates with confidence intervals at ρ=0 for the ACME, average direct effect (ADE), and total effect. ACME₀(ρ) and ACME₁(ρ) respectively represent average causal mediation effects under control and treatment conditions. Mediation effects are considered robust when confidence intervals exclude zero across a reasonable range of ρ values (typically ±0.5).

**Key Findings:** Physical fatigability's mediation effect on the loneliness-sarcopenia relationship remained statistically significant across a reasonable range of ρ values, suggesting relative insensitivity to unmeasured confounding. For mental fatigability, mediation effects approached significance for muscle mass (ρ between -0.3 to 0.3) but remained non-significant for overall sarcopenia.
